# Acknowledgement to Reviewers of *Children* in 2018

**DOI:** 10.3390/children6010009

**Published:** 2019-01-11

**Authors:** Children Editorial Office

**Affiliations:** MDPI AG, St. Alban-Anlage 66, 4052 Basel, Switzerland

Peer review is an essential part in the publication process, ensuring that *Children* maintains high quality standards for its published papers. In 2018, a total of 166 papers were published in the journal. Thanks to the cooperation of our reviewers, the median time to first decision was 28 days and the median time to publication was 44 days. The editors would like to express their sincere gratitude to the following reviewers for their time and dedication in 2018:
Aadland, EivindLange, JoannaAaron, RachelLau, EricaAbbeddou, SouheilaLeBovidge, JenniferAhmed, Atif AliLeis Trabazo, Maria RosauraAkbarfahimi, MalahatLiller, KarenAlam, MohammadLin, Pei-YiAllen, TarynLin, Yuan-ChiAlsharairi, NaserLinhares, MariaAmiram, NirLittlewood, Robyn A.Anbar, RanLiu, YanlingAnderssen, SigmundLiu-Smith, FengApplebaum, MarkLøken, Elin B.Ardigo, LucaLópez-López, DanielArnbjörnsson, EinarLotstein, DebraArteaga, OlatzLu, Tsung-HsuehAshish, PathakLucey, AliceAvez-Couturier, JustineLuick, BretAyonrinde, OyekoyaMair, JacquelineBadau, DanaMakena, MonishBadicu, BadicuMalby Schoos, Ann-MarieBaker, EmmaMandrell, BelindaBalagopal, BabuMarchesi, AlessandraBarbi, EgidioMarom, TalBarratt, MichaelMartin, SarahBax, KarenMartino Alba, RicardoBender, Hans-UlrichMartins, NatáliaBennett, William E.Martucciello, GiuseppeBerger, StuartMascherini, GabrieleBergstraesser, EvaMaslin, KateBhabha, JacquelineMattei, PeterBianchetti, Mario G.Mavilidi, MyrtoBoddy, Lynne M.Mayer, AntonBoles, Jessika C.McKinlay, AudreyBooth, JosieMcKinnon, Rachel D.Boss, ReneeMcLeod, Lisa M.Bravaccio, CarmelaMcLoney, Eric D.Breuner, CoraMcManus, AliBriles, David E.Meendering, JessicaBrønd, JanMeyer, Rosan W.Brooks, Audrey J.Meyerholz, David K.Brown, HelenMherekumombe, Martha F.Brusseau, TimMiller, Elissa G.Buchowski, MacMisra, SanghamitraBurgess, John A.Mooney-Doyle, KimBurr, LucyMoore, TimCadell, SusanMorales Suárez-Varela, María M.Cahova, MonikaMoritz, Michael L.Caldwell, CurtisMorrison, Wynne E.Camacho Miñano, MaríaMorton, PaulCamenga, Deepa R.Moser, DominikCarey, CliftonMuñoz-López, FranciscoCarlin, AngelaMurphy, AndrewCarotenuto, MarcoNandi, DeipanjanCarter, Brian S.Nelson, SarahCasanovas, CarmenNeri, CaitlinCatlin, AnitaNicolini, AntonelloCentofanti, Stephanie A.Nilsson, StefanChang, Kay W.Nishimura, NoriyukiChang, Mary P.Norris, EmmaChen, Kong Y.Nosetti, LuanaCheng, JiaNuttall, JoceChhatwal, JugeshO’Grady, LynChristoph, MaryOkajima, HideakiChruściel, PawełOkhuysen-Cawley, ReginaComan, EmilPalmer, Ruth H.Cools, WouterPan, LuCorpeleijn, EvaParadies, YinCotton, Robin T.Pavone, PieroCozzi, GiorgioPbert, LoriCrenn, PascalPendergrast, RobertCriss, ShaniecePentaris, PanagiotisCrock, MaryPesola, Arto J.Cruz, MariaPeters, Rachel L.Cuberos, RamónPeterson, CatherineCunningham, MelodyPeyer, KarissaDampier, CarltonPhillips, FaryaDanysh, Heather E.Pilishvili, TamaraDe Cock, NathaliePillai Riddell, RebeccaDe Müllenheim, Pierre-YvesPokorska-Śpiewak, MariaDe Salazar-Torres, Jose JesusPostier, AndreaDeFilippis, MelissaPozzobon, MichelaDenney-Koelsch, Erin M.Prince, BennieDesha, Laura N.Prout Parks, ElizabethDing, CodyPuertas Molero, PilarDore-Stites, DawnPuri, KritiDowning, JuliaRachele, JeromeDrake, RossRandall, CameronDuckham, BryanRea, CorinnaDudeney, JoanneReal, Francis J.Edirippulige, SisiraRevelli, LucaEhsan, ZarminaRice, AlexandraElls, LouisaRigante, DonatoEndersby, RaeleneRiley, NicholasEsparham, AnnaRinaldo, NatasciaEun, Ki-SooRoberson, Donald N.Faber, Anthony C.Robert, LidiaFadda, RobertaRobitaille, EricFairclough, StuartRocha-Ferreira, EridanFalup-Pecurariu, OanaRodin, GaryFeraco, AngelaRogobete, Alexandru FlorinFernández-Revelles, Andrés B.Rosen, LawrenceFineberg, IrisRoss, Alyson C.Fitzpatrick, EmerRossignol, Daniel A.Flores-Huerta, SamuelRuben, LewisFradkin, ChrisRussell, Alexandra C.Gardner, ReneeRutkauskaite, RenataGaretz, SusanRyabov, IgorGartner, Lawrence M.Ryder, Justin R.Geraldo, AndreiaSaad, MarceloGerbershagen, MarkSamadi, Sayyed AliGershan, LynnSamsel, ChaseGevirtz, RichardSantos, MariaGeyer, James D.Sato, MarikoGianfrancesco, Milena A.Sayón-Orea, CarmenGillett, Emily S.Schafer, Ellen J.Gilliland, JasonSchechter, NeilGodbout, RogerScherzer, AlfredGogou, MariaSchwaberger, BernhardGoh, TanSetty, BhuvanaGoldfarb, SamuelShah, NilayGolianu, BrendaShin, JaewookGollop, MeganShyman, EricGondi, Christopher S.Sibinga, EricaGonzález-Stuart, ArmandoSiegel, Robert M.Good, PhillipSil, SoumitriGraf, NorbertSimon, PatrickGray, AndrewSimons, JoanGulliver, PaulineSindher, SayantaniHa, PatrickSiniscalco, DarioHain, RichardSjöström, ElisabethHainsworth, KeriSoma, CaelanHamblin, RebeccaSpiegler, JulianeHamilton, ThomasSpraker-Perlman, HollyHan, Yueh-YingSrivastava, GitanjaliHand, Ivan L.Stewart, LauraHashimoto, DanielStone, AmandaHauer, JulieSubnis, UtkarshHeise, CarlosSulman, Cecille G.Hemphill, JeanTachtsidis, IliasHendren, RobertTannuri, Ana CristinaHerbert, AnthonyTeo, AndrewHolmes, Geoffrey K.Teschke, RolfHoltzen, HollyThompson, DeanneHori, HirokiTonini, GianHosie, AnnmarieTutor, James D.Houtchens, MariaUbago, Jose LuisHsin, JenniferUchida, HajimeHuertas-Delgado, FranciscoUrbanelli, LorenaHughes, Jean R.Van Der Ende, Peter C.Humphrey, LisaVargas, Jorge H.Humphrey, LisaVarshney, PoojaIaffaldano, PietroVasickova, JanaIde, KazukiVazou, SpyridoulaIkeda, ErikaVeatch, Olivia J.Irtan, SabineVenalainen, TaisaJacob, JennaVeneri, Diana A.Jahnel, JörgVesoulis, Zachary A.Jamar, GiovanaVinall, JillianJang, SiwonWadhwaniya, ShirinJayatissa, RenukaWager, JuliaJensen, RoxanneWainwright, NaldaJoronen, KatjaWake, MelissaJou, HsingWang, JianxiongKaiser, PamelaWanigatunga, Amal AsiriKaiser, TillWatanabe, EiichiKang, Jae-HeonWeaver, MeaghannKarlson, CynthiaWebb, LindseyKavanagh, PatriciaWeiser, DanKemper, Han C.G.Weiss, AnnetteKemper, KathiWilson, KimKhan, Saira A.Wilson, Thomas J.Klein, MarkusWitkamp, FrederikaKöglmeier, JuttaWoodfield, LorayneKohyama, JunWu, FeitongKovacic, Katja K.Wyver, ShirleyKovesi, TomYang, WaiKrüger, CarstenYiu, EppieKulaga, ZbigniewYourkavitch, JenniferKumar, RamYuan, HongKumar, SeemaZhou, DongLachat, CarlZurita Ortega, FélixLaird, YvonneZurynski, Yvonne

